# A randomized clinical trial of mindfulness training versus a health promotion program: Impact on cognitive and mental health in older immigrants

**DOI:** 10.1016/j.ijchp.2025.100642

**Published:** 2025-10-24

**Authors:** Ana C. Teixeira-Santos, Leandro Gomes, Diana R․ Pereira, Fabiana Ribeiro, Joana Carvalheiro, Catarina Godinho, Anabela Silva-Fernandes, Etienne Le Bihan, Carine Federspiel, Jean-Paul Steinmetz, Anja K․ Leist

**Affiliations:** University of Luxembourg, Luxembourg

**Keywords:** Older adults, Mindfulness-based interventions, Health promotion program, Cognition, Mental health, Social determinants of health, Immigrant health

## Abstract

**Background:**

Mindfulness-based stress reduction (MBSR) has shown benefits for cognition and stress relief. Enhancing these functions may have a protective role in vulnerable populations, particularly older immigrants who face a higher risk of neurodegenerative disease. However, whether MBSR can have positive effects on cognitive and affective functions in these populations remains understudied. This trial compared the effects of MBSR with a health promotion program (HPP) in older immigrants.

**Methods:**

In this single-center, randomized, double-blind controlled trial, 151 Portuguese-speaking older immigrants (≥55 years old) residing in Luxembourg were screened and 89 participants (age range: 55–80, *M* age: 62.58 years ± 6.08, 72 % women) were randomized to 2-month weekly group interventions of either MBSR (*n* = 44) or HPP (*n* = 45). Data were collected at three time points: baseline, immediately after the intervention (post-intervention), and at follow-up, conducted one to three months after the intervention. Executive functioning measures, including Letter-Number Sequencing, Trail Making Test, and Stroop color-word, were the main outcomes. Secondary outcomes included general cognitive functioning, cortisol level, heart rate variability, and self-reported affective and mindfulness states.

**Results:**

75 % of participants in the MBSR group and 53 % in the HPP group completed at least one post-assessment. Linear mixed model analyses showed significant time effects in Letter-Number Sequencing (*p* = .04), as well as reductions in anxiety (*p* < .01) and perceived stress (*p* < .01), with no significant group differences or group × time interactions. These improvements were observed from baseline to post-intervention and still persisted at follow-up.

**Conclusion:**

Both interventions positively influenced attention, with the most notable improvements observed in anxiety and perceived stress. These findings suggest that group interventions may have the potential to improve cognitive and affective indicators, regardless of their specific content. Despite their diverse goals, the interventions shared procedural features, such as the organization and delivery of the sessions, which may have contributed to the outcomes observed. This underscores the potential value of well-designed group-based programs in cognitive and affective indicators among vulnerable older adults. While further research is needed, our findings point to the relevance of including these interventions within the realm of promoting healthy aging and dementia prevention.

## Introduction

Aging is a significant concern in modern society, with the aging population facing increased risks of cognitive decline and neurodegenerative diseases (World Health Organization, 2022). Immigrants, particularly older adults, represent a population at heightened risk due to various factors such as acculturation, stress, language barriers, and socioeconomic disparities (Kivipelto et al., 2018; Kristiansen et al., 2016). Lifestyle interventions, including stress reduction techniques and multidomain interventions, have emerged as promising strategies to mitigate cognitive decline and improve overall well-being in aging (Isenberg, 2020).

One such intervention gaining traction is mindfulness-based practices. Mindfulness, as defined by Kabat-Zinn (1990), involves purposefully paying attention to the present moment without judgment. In mindfulness sessions, practitioners train to stay in the present moment by focusing their attention on specific stimuli, such as their breath, while cultivating awareness of their thoughts, emotions, and bodily sensations. This practice is believed to enhance skills related to sustained attention and executive control, making it beneficial for cognitive functioning (Yakobi et al., 2021). The rationale behind this is that mindfulness activities require attentional resources such as maintenance of information and monitoring of attention, which are related to working memory skills (Jha et al., 2019). Studies have also explored the mechanistic pathways through which mindfulness training influences cognitive processes (Barrett et al., 2019; Hölzel et al., 2011b). For example, brain imaging studies have shown that mindfulness training can increase connectivity between brain areas critical for memory and executive functioning (Sevinc et al., 2021; Yue et al., 2023) and promote plasticity changes (Hölzel et al., 2011a).

Additionally, mindfulness-based interventions have been shown to heighten self-attributed mindfulness (Brand et al., 2012) and alleviate anxiety and depression (Li et al., 2024; Ong et al., 2012; Schreiner & Malcolm, 2008; Wetherell et al., 2017), while also improving sleep quality (Brand et al., 2012; Perini et al., 2023; Wetherell et al., 2017). Furthermore, research suggests that mindfulness may reduce stress levels, as evidenced by changes in physiological parameters such as cortisol levels. Specifically, mindfulness interventions have been associated with a decrease in morning cortisol levels (Brand et al., 2012). Heart rate variability (HRV), which refers to variations in heart rate from one beat to the next, also serves as a physiological measure of autonomic nervous system function. In mindfulness research, HRV is used as an indicator of emotional regulation (Brown et al., 2021), with higher HRV associated with greater resilience to stress (Appelhans & Luecken, 2006).

Given the potential effects of mindfulness on cognitive and affective indicators, older individuals from vulnerable populations, who are at higher risk of developing dementia, could, in principle, benefit from it. However, the evidence for positive sustained effects of mindfulness techniques remains mixed, especially in the studies with active comparators, showing no significant effects (Whitfield et al., 2022). Furthermore, few studies focused on vulnerable older populations [see Mirabito and Verhaeghen (2022) for a review] and most research has involved younger participants (Cásedas et al., 2020; Im et al., 2021; Khoury et al., 2015; Lao et al., 2016; Sharma & Rush, 2014; Winbush et al., 2007). Prior meta-analyses indicate that while mindfulness-based interventions demonstrate superiority over control conditions in older populations, particularly in enhancing executive function, it appears not effective in younger adults in improving attention, declarative memory, or overall cognitive aging (Whitfield et al., 2022). However, in older adults with subjective cognitive complaints but not dementia, mindfulness showed no superior effect on episodic memory, executive function, structural brain changes, functionality, or self-reported cognitive measures at 6- or 18- month timepoints when compared to physical exercise and health education control (Lenze et al., 2022). Hence, there is a critical need for more comprehensive, rigorous investigations into the impact of mindfulness practices on aging.

Furthermore, it is important to point out that the field faces methodological challenges, as many previous studies lack a control condition or use only a passive control (see Baer, 2003; Gard et al., 2014; Gill et al., 2020; Johnson, 2023). However, an active control condition is crucial to account for the Hawthorne effect, which refers to participants changing their behavior simply because they are aware of being observed (Adair, 1984). To address these challenges, the MEDITAGING study aimed to compare cognitive, affective, and physiological outcomes of two group-based programs, the Mindfulness-based Stress Reduction (MBSR) and a health promotion program (HPP), targeted at Portuguese-speaking immigrants aged 55 and above, residing in Luxembourg and neighboring regions.

By incorporating an active control condition, we hypothesized that participants in both intervention groups would experience benefits in cognitive, affective, and physiological measures (Fountain-Zaragoza & Prakash, 2017; Gard et al., 2014; Gill et al., 2020; Pandya, 2020). Importantly, we predicted that the MBSR group would exhibit greater improvements in executive functioning (primary outcome), consistent with the program's aims (Kabat-Zinn, 1990). In a similar fashion, we predicted that the MBSR group would exhibit greater stress reduction, as measured by self-reported questionnaires, cortisol levels (Wetherell et al., 2017), and HRV (Nijjar et al., 2014), as well as larger improvements in anxiety and depression (secondary outcomes).

## Methods

### Study design

MEDITAGING is a single-center, randomized, double-blind controlled superiority clinical trial consisting of two parallel arms: one group received the MBSR program, while the other underwent the HPP, an active control condition with no mindfulness. The study protocol was previously registered in ClinicalTrials.gov (Registration number: NCT05615337) and published (Teixeira-Santos et al., 2023).

**Fig. 1:**
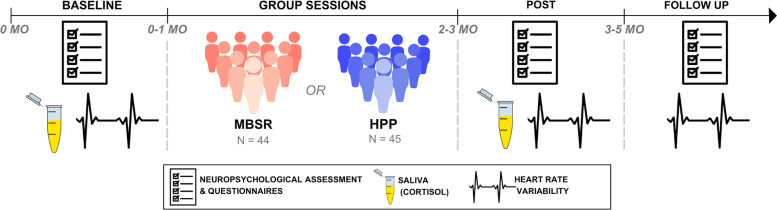
Study design.

Participants were randomized into these two conditions after a baseline assessment. Nine group sessions were conducted in person, with class sizes ranging from six to 14 participants, averaging 9.89 ± 2.98 participants per group. The intervention lasted two months, with individual assessments conducted at three time points: baseline, post-intervention, and follow-up, which occurred one to three months after the end of the intervention. The assessments and group sessions were held in a quiet and private room at the facilities of the collaborator institutions such as senior public services and recreational clubs in Luxembourg. Please, refer to Fig. 1 for the study design.

Mindfulness-Based Stress Reduction (MBSR, shown in red); Health Promotion Program (HPP, shown in blue). Assessments were conducted in the same individualized manner in all sessions, on different dates for each participant. Flexibility in scheduling was maintained to accommodate participants' frequent travels to their country of origin.

Ethical approval was obtained from the Ethics Review Panel of the University of Luxembourg (ERP 21–042 MEDITAGING), the National Research Ethics Committee (CNER - 0422–89), and the Luxembourg Ministry of Health. Written informed consent was obtained from all participants.

### Participants

A total of 151 participants were recruited in Luxembourg from May 2022 to April 2023, with the last follow-up in September 2023. Recruitment came to a halt in March 2023 due to time constraints, with one participant still required to reach the intended sample size of 90 participants (for details on sample size calculation, see section below). The recruitment strategies encompassed press channels (e.g., newspapers, radio programs), web-based platforms (e.g., social media, websites), printed flyers, talks at local community events (e.g., churches), and direct contact with participants and Portuguese-speaking associations, local clinics, and senior clubs.

To be eligible, individuals had to be 55 years old or older, Portuguese-speaking immigrants, residing in the Grand-Duchy of Luxembourg or the Greater Region, and free from dementia. During the screening phase, we used the Portuguese adaptation of the Mini Mental State Examination (MMSE) for dementia screening (Santana et al., 2016). A MMSE cutoff score of 22 was used, as most participants were expected to have a low education level (Kochhann et al., 2010). Other exclusion criteria included the presence of severe psychiatric conditions, assessed by DSM-5 Self-Rated Level 1 Cross-Cutting Symptom (Bastiaens & Galus, 2018), severe medical conditions, impairment of vision, hearing, or communication abilities, and engagement in mindfulness-based activities, cognitive training, or other psychological interventions within the past three years. These conditions were evaluated through interviews. Illiterate participants were excluded as reading and writing were essential for participation in both groups. Participants received no compensation for their participation in the study.

### Sample size calculation

No previous research had investigated the cognitive impacts of MBSR specifically in older migrant adults, at the time we designed the study. However, prior research involving older adults indicated a modest effect size of MBSR on attention after intervention (Cohen’s *d* = −0.24) (Moynihan et al., 2013). Based on these findings, we anticipated a small effect (*f* = 0.14). Using the G* Power – 3.1 statistical software (Faul et al., 2007) for repeated-measures ANOVA, focusing on within-between interaction (group x time interaction), with 80 % power and 5 % type I error rate, we computed the sample size estimation. Considering a 5 % attrition rate observed in Moynihan et al. (2013), a total of 90 participants was estimated, evenly split with 45 in each group, as planned in the pre-registered protocol (Teixeira-Santos et al., 2023).

### Randomization and masking

We randomly assigned participants into the MBSR or the HPP in a 1:1 ratio, using a computer-generated allocation list previously generated from a website (http://www.randomization.com), by the primary author (Ana C. Teixeira Santos – ACT), in blocks of ten (five individuals randomly allocated to each group). The allocation list was concealed from all study personnel, with each condition recorded in separate Excel sheets. ACT was responsible for enrolling participants and only had access to their assigned condition after the baseline assessment was completed, at which point she communicated the group session information to the participants. While participants were aware of their assigned activities, they did not know whether they were in the experimental or control group. Assessors were kept unaware of the conditions. After randomization, assessments were not conducted by the individual who led the intervention (ACT). Participants’ blinding was assessed using a credibility and expectancy questionnaire (Devilly & Borkovec, 2000) at the conclusion of the group sessions. Outcome assessors were blinded to allocation, but the intervention facilitator (ACT) and data analysts (ACT and JC) were not blinded.

#### Interventions

Sessions were delivered weekly in face-to-face groups of approximately ten individuals, each lasting 2.5 h. The MBSR adhered to the original MBSR curriculum (Kabat-Zinn, 1982; Santorelli et al., 2017), consisting of eight weekly sessions, along with a half-day retreat of four hours. The MBSR integrates secular mindfulness meditation practices into a structured program designed to mitigate distress through self-regulation mechanisms. Participants were trained to cultivate mindfulness, involving attention to the present moment and awareness of their thoughts, emotions, and bodily sensations. The program incorporated various techniques, including guided body scan, hatha yoga, and diverse meditation practice (e.g., sitting, eating, and walking). The program also included discussions on stress physiology and coping strategies, with daily at-home practice. Participants were encouraged to engage in informal mindfulness practices in their daily activities, such as performing one activity mindfully each day. Towards the end of the program, a half-day-silent retreat was provided to further develop meditation skills using various techniques, allowing participants to engage in introspection for an extended period.

The HPP consisted of a psychoeducation program focused on health literacy and habit development, based on existing literature (Livingston et al., 2020; Lorig Kate et al., 2020; MacCoon et al., 2012; Mace et al., 2022), covering topics like risk factors for dementia, sleep quality, nutrition, addressing unhealthy behaviors, physical activity, and cognitive stimulation. Each session was carefully planned with a predefined structure, and it was meticulously tracked by the instructor across all groups.

Both interventions were aligned in terms of setting and duration to ensure balanced comparisons, which also included the 40-min at-home daily exercises. Following each session, participants engaged in discussions about their experiences and how they have incorporated the practices into their daily routines. Participants received a weekly manual containing the covered topics, instructions for home exercises, and accompanying audio materials. For more details about the interventions, see Teixeira-Santos et al. (2023).

Both groups' sessions were facilitated by the primary author, ACT, who is a psychologist qualified to administer the MBSR program by the UC San Diego Center for Mindfulness. ACT has extensive experience and expertise in group interventions, community therapy, teaching, and a background in the Psychology of Aging. One of the other co-authors (FR) conducted one of the HPP sessions (the watercolor session).

#### Outcomes

The assessment included standard neuropsychological tests and self-report questionnaires covering various aspects, including participant sociodemographic and health, medical history, alcohol and drug consumption, physical activity levels, engagement in meditation or other therapeutic activities, perceived stress, and sleep quality. Three different assessors were trained to conduct the assessment in a standardized manner.

#### Primary outcomes

The primary outcomes focused on executive functioning, which were assessed using the following measures:

(1) the **Letter-Number Sequencing from the Wechsler Adult Intelligence Scale** (WAIS-III; Wechsler, 2008) was used to evaluate working memory. The Portuguese version has demonstrated good psychometric properties, with an internal consistency of *r* = 0.84, using the split-half reliability method. The correlation across applications was 0.71, indicating good temporal stability. Additionally, its structural validity was confirmed in older adults (Pezzuti & Rossetti, 2017). The outcome measure was the number of correct sequences (Sánchez-Cubillo et al., 2009).
(2) the **Trail Making Test** (TMT; Cavaco et al., 2013) serves as a direct indicator of executive control abilities (Sánchez-Cubillo et al., 2009). The TMT has been widely used to assess this construct and has demonstrated robust temporal reliability in Portuguese-speaking individuals, with an Intraclass Correlation Coefficient (ICC) of 0.91 for Part B, using the test-retest method (Batista et al., 2013). The interference score, calculated as the difference in execution time between Part B and Part A, was used, with higher scores indicating worse performance.
(3) the **Stroop Color-Word Test** (Golden, 1978) is a widely used measure to assess cognitive interference and executive function. In the context of Portuguese older adults (see Fernandes, 2013), the test demonstrated a Cronbach’s alpha of 0.66, indicating acceptable internal consistency. It also exhibited temporal stability, with reliability coefficients typically exceeding 0.71. Furthermore, a factorial structure comprising eight factors was identified, accounting for 96.16 % of the total variance (Fernandes, 2013). In our study, the interference score was calculated as the difference between the number of color-words (CW) correctly named and the estimated score (CW') for this task. Higher scores indicate worse performance. The formula used was:
  Interference score = *CW – CW’*
  $${\text{CW}}^{\prime }=\frac{\text{Number}\;\text{of}\;\text{words}\;\text{read}\;\text{correctly}+\text{Number}\;\text{of}\;\text{colors}\;\text{named}\;\text{correctly}}{\;Number\;of\;words\;read\;correctly\times Number\;of\;colors\;named\;correctly}$$

#### Secondary outcomes

Secondary outcomes included:

(1) The **MMSE** was used to assess general cognitive ability, including tasks related to orientation, retention, attention and calculation, delayed recall, language, and visuoconstructive ability. The choice of this test was due to its relatively simple items, which is particularly important given that our sample consists of individuals with low educational attainment. The MMSE is the most widely used cognitive screening worldwide and has demonstrated moderate-to-high internal consistency, satisfactory test-retest reliability, and good convergent validity (Tombaugh & McIntyre, 1992). Normative data for the Portuguese population were developed using a representative sample of 850 cognitively healthy adults from the Portuguese community (Freitas et al., 2015). The MMSE has also been evaluated in Portuguese individuals with mild cognitive impairment and various types of dementia (e.g., Alzheimer’s disease, vascular dementia, frontotemporal dementia). The test showed excellent specificity and sensitivity for Alzheimer’s disease, although it presented limited diagnostic accuracy in detecting MCI and other types of dementia (Santana et al., 2016). The score corresponds to the sum of correctly answered items, with higher MMSE scores indicating better general cognitive functioning.
(2) The **Pittsburgh Sleep Quality Index** (PSQI; Del Rio João et al., 2017) is a self-rated questionnaire designed to assess sleep quality and disturbances over a one-month period. It consists of 19 items that generate seven component scores: subjective sleep quality, sleep latency, sleep duration, habitual sleep efficiency, sleep disturbances, use of sleep medication, and daytime dysfunction. The validation of the PSQI for the Portuguese population (Del Rio João et al., 2017) revealed a single-factor structure with good factor loadings for all items, supporting the scale's construct validity. Additionally, the PSQI demonstrated an adequate degree of internal consistency, with a Cronbach's alpha coefficient of 0.70 and it effectively differentiates between good and poor sleepers. In our study, we used the global PSQI score, with a value greater than 5 indicating poor sleep quality.
(3) The **Perceived Stress Scale** (PSS; Cohen et al.Mota-Cardoso et al., 2002, 1983) is a self-report measure designed to assess the degree to which individuals perceive situations in their lives as stressful. It evaluates how unpredictable, uncontrollable, and overloaded individuals find their lives during the past month. The Portuguese version (Mota-Cardoso et al., 2002) consists of 10 items rated on a Likert scale, with higher total score indicating greater levels of perceived stress. The PSS demonstrates good internal consistency and validity, with a two-factor structure predominance (Lee, 2012). There is also evidence supporting its validity in older populations (Ezzati et al., 2014).
(4) The Geriatric Depression 5 Item Scale (GDS; Santos et al., 2019) is a screen measure for depressive symptomology particularly developed for older populations. The short version was used to reduce assessment fatigue. In Portuguese older adults, it shows a Cronbach’s alpha of 0.69, with acceptable sensitivity (70 %) and specificity (85 %). However, the confirmatory factor analysis does not support the one-dimensional structure of this version (Santos et al., 2019).
(5) The **Geriatric Anxiety Inventory** (GAI, Ribeiro et al., 2011) is a measure developed to assess anxiety symptoms in older adults. The Portuguese adaptation was tested considering a community-dwelling sample of older and clinical subgroups of outpatients, namely people meeting the criteria for an anxiety disorder, depressive disorder, or early Alzheimer’s disease (Ribeiro et al., 2011). The Portuguese version demonstrates strong discriminatory ability between the different groups. Regarding reliability, this instrument yielded a good internal consistency (Cronbach’s alpha was 0.96) and test-retest reliability (intraclass correlation coefficient of 0.96). With respect to validity, the results showed good concurrent validity (between 0.63 and 0.86) and satisfactory specificity and sensitivity indicators when using the threshold score of 8/9.
(6) The **Mindfulness Attention and Awareness Scale** (MAAS; Gregório & Pinto-Gouveia, 2013) was employed to assess the mindfulness trait. The self-report scale consists of 15 items rated on a 6-point Likert scale, with higher scores indicating greater levels of mindfulness. The Portuguese adaptation (Gregório & Pinto-Gouveia, 2013) has demonstrated robust psychometric properties. It exhibits high internal consistency, with a Cronbach’s alpha of 0.90 and strong convergent validity. Confirmatory factor analyses of this version revealed a single-factor solution with 14 items, showing good model fit. Furthermore, cross-validation procedures confirmed the measurement invariance across two independent samples from the general population, underscoring its robustness.
(7) Physiological measures (i.e., **salivary cortisol** and **HRV**) were also assessed as secondary measures. Saliva samples were collected by the participants at baseline and after the last group session, but not during the follow-up assessment. To calculate an estimate of the overall cortisol released, it was determined the area under the concentration-time curve of daily cortisol levels (ng/ml), which were sampled at 0, 30, and 480 min after waking. More specifically, cortisol levels were derived by the area under the curve above the baseline value minus the area above the curve below the baseline value (AUC_I_) (for the rationale for this calculation see Fekedulegn et al., 2007). HRV was individually recorded at the beginning and at the end of each of the three assessment sessions and an average between these two measures was calculated for each assessment session. Data was recorded using a Polar H10 chest strap (Polar Electro, Kempele, Finland) positioned beneath the participants' pectoral muscles and transmitted to the Elite HRV app. Participants were instructed to sit in a comfortable position with their eyes closed for a duration of 10 min. We utilized the Elite HRV-derived values, automatically provided by the app, for the root mean square of successive differences (RMSSD) over the entire recording period (Kim et al., 2018).

Adverse effects were assessed through the Positive and Negative Affect Schedule (PANAS) and a Visual Analog Scale (VAS), which was specifically designed to measure various subjective experiences such as fatigue, anxiety, sadness, sleepiness, depression, pain, stress, low self-esteem, confusion, loss of appetite, loneliness, distraction, agitation, feeling devalued, feeling overwhelmed, and reliving traumatic memories. Participants were asked to indicate the intensity of these feelings either at the present moment or during the previous week. These assessments were administered in five group sessions and complemented by open questions during post-intervention interviews. For a broader description of each outcome see Teixeira-Santos et al. (2023).

### Adherence and dropout rate analysis

For the adherence and dropout rate analysis, we considered the proportion of participants who had completed the baseline assessment and attended the first group session, as well as the number of participants who attended at least three group sessions.

### Statistical analysis

The modified intent-to-treat analysis included all participants who attended at least three of the nine intervention sessions and at least one of the post-assessment sessions (either the assessment session immediately following the intervention or the follow-up session one or three months later), unlike the strict intent-to-treat approach which includes all participants originally allocated regardless of their adherence to the intervention. This modified approach accounts for participants who engaged minimally in the intervention, providing a realist measure of its efficacy (Armijo-Olivo et al., 2009).

Analyses did not involve the exclusion of any outliers and no imputation was performed (see supplementary material for additional information). Categorical variables were described using frequencies and percentages, while continuous data were summarized with means and standard deviations (*SD*).

Statistical analyses were conducted using linear mixed-effects models for each of the primary and secondary outcomes. These analyses were performed with Rstudio (Kuznetsova et al., 2017), version 2023.06.0 Bild 421 (R Core Team, 2018), utilizing the 'lmer' function from 'lme4′ package (Bates et al., 2015). In these models, the interaction between group and time, as well as their main effects, were included as fixed effects, with participants specified as a random effect. Additionally, sensitivity analyses were conducted using the same model while controlling for age, sex, education, and number of sessions attended to verify whether the results remained consistent.

We assessed the interaction between group and time separately for each of the primary and secondary outcomes. If the interaction was significant, main effects were not interpreted and we further explored the direction of the effect by performing post-hoc pairwise comparisons (with Tukey adjustment method to control for multiple comparisons), using the 'emmeans' R function. Significant effects were set at *p* < .05. When the interaction was not significant, but a main effect of time or group was found, we further assessed the direction of the main effect by fitting a new linear mixed-effect model with only time or group as fixed effect (participants were included as a random effect as in the main model) and conducted pairwise comparisons between the different time points or groups using the ‘lsmeans’ R function.

Following the procedure described by Perini et al. (2023), Hedges’*g* effect sizes (Hedges, 1989) were calculated for all scores though bootstrap resampling techniques (Ho et al., 2019), using DABEST toolbox in MATLAB (https://github.com/ACCLAB/DABEST-Matlab). Effect sizes were computed separately for each group, comparing the baseline performance to post-intervention and follow-up sessions.

Additional analyses of Spearman correlation were conducted to investigate whether gains in the significant primary outcomes identified in the analyses above were correlated with individual factors such as educational level, number of attended sessions, sex, perceived stress, depression, mindfulness trait, anxiety, cortisol level, and sleep quality. Gains in outcomes were calculated as the difference between baseline and post-intervention scores. By examining these correlations, we sought to examine potential patterns that could inform future interventions and enhance our understanding if these factors interact with the effects of the group programs on cognitive and affective outcomes.

## Results

**Fig. 2:**
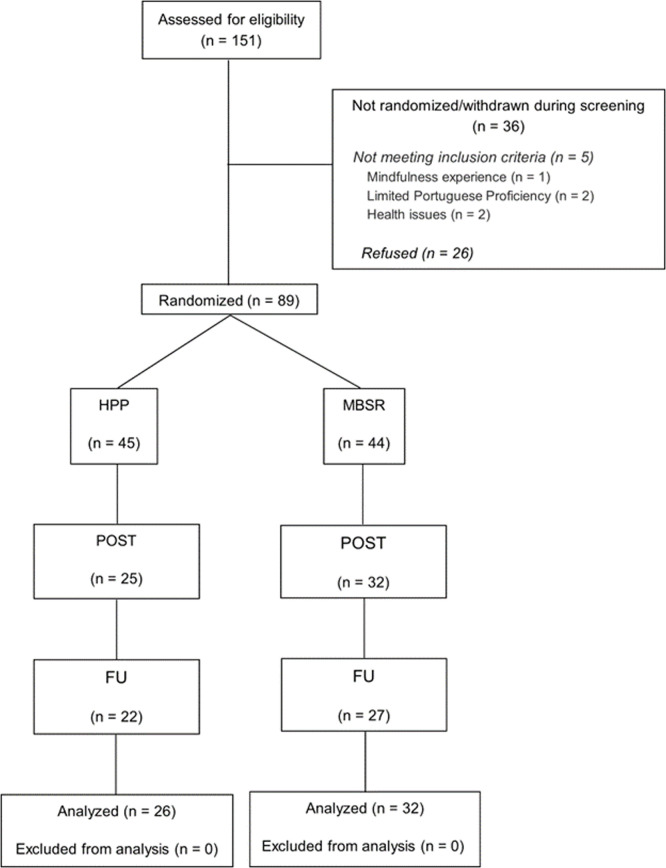
Participant flow diagram.

We used the CONSORT checklist for randomized trial when writing our report (Schulz et al., 2010). Fig. 2 illustrates the participant flow diagram following the CONSORT guidelines. Initially, 151 individuals were screened, of which 89 (average age: 62.51 years ± 6.28, 63 [70.8 %] women) were randomly allocated to either the Mindfulness-Based Stress Reduction (MBSR, *n* = 44) or the Health Promotion Program (HPP, *n* = 45) groups, to be assessed across three time points: (1) baseline; (2) post-intervention; and (3) follow-up. At baseline, both groups were comparable across all measured variables (refer to Table 1).

The total number of individuals approached during the recruitment phase was not documented. Some participants did not complete the post-intervention assessment session (POST) but did participate in the follow-up session (FU). In such cases, these participants were included in the analysis. All analyses were conducted by original assigned groups. MBSR, Mindfulness-Based Stress Reduction; HPP, Health Promotion Program.

**Table 1:** Sociodemographic and health characteristics between groups at baseline.

	HPP (*n* = 45)	MBSR (*n* = 44)	*p*
Age	63.73 (6.78)	61.41 (5.08)	.09
Sex, No.			
*Women*	31 (68.9 %)	33 (75.0 %)	.52
*Men*	14 (31.1 %)	11 (25.0 %)	
Lifelong Occupation^1^^1^			.30
*Level I* *Level II* *Level III* *Level IV* *Level V*	0.00 (0.0 %) 1.00 (2.2 %) 4.00 (8.9 %) 9.00 (20.0 %) 31 (68.9 %)	0.00 (0.0 %) 0.00 (0.0 %) 1.00 (2.3 %) 13 (29.6 %) 30 (68.2 %)	
Relationship status			.90
*Single* *Married* *Separated* *Divorced* *Widowed*	2 (4.4 %) 24 (53.3 %) 1 (24.4 %) 11 (24.4 %) 7 (15.6 %)	2 (4.5 %) 25 (56.81 %) 0 (0.0 %) 11 (25.0 %) 6 (13.6 %)	
Current smoker	4 (8.9 %)	4 (9.1 %)	.63
Years of formal education	7.38 ± 4.19	7.59 (3.40)	.33
BMI, M (*SD*)	27.70 ± 5.52	27.42 (3.4)	.62
Country of Origin			.16
*Angola* *Brazil* *Cape Verde* *Guinea-Bissau* *Portugal* *São Tomé and Príncipe*	1 (2.2 %) 0 (0.0 %) 1 (2.2 %) 3 (6.7 %) 40 (88.9 %) 0 (0.0 %)	0 (0.0 %) 1 (2.3 %) 6 (13.6 %) 1 (2.3 %) 35 (79.5 %) 1 (2.3 %)	
Length of residence in Luxembourg (years)	28.84 (14.51)	24.39 (13.85)	.14

Continuous variables were analyzed using independent *t*-tests or Mann-Whitney tests when normality assumptions were not met. Categorical variables were compared using chi-square tests. Continuous variables are presented as *M* (*SD*) and categorical variables as frequency ( %).

Abbreviations: BMI, Body Mass Index; BSL, baseline session; POST, post-intervention session; FU, follow-up session.

1 Classification adapted from Graffar (1956) comprising five levels, from I (highest) to V (lowest).

Thirty-two (72.7 %) participants in the MBSR group and 26 (57.8 %) in the HPP group completed at least one post-intervention assessment (post-intervention or follow-up). In total, 31 (34.8 %) participants discontinued the study, resulting in 57 (64.0 %) participants completing the post-intervention session and 49 (55.1 %) completing the follow-up session. The analysis included all participants who attended at least three intervention sessions and one of the post-intervention assessment sessions, totaling 58 participants, equivalent to 65.2 % of those initially enrolled. No differences were identified between completers and dropout participants in terms of age, education, general cognitive functioning, sex, smoking habits, and body mass index (refer to Supplementary Table S1). Additionally, there was no significant difference in dropout rates between the HPP (*n* = 19) and MBSR (*n* = 12) groups, *χ*²(1) = 2.19, *p* = .14. Additionally, the number of days between the assessment sessions (baseline to post-intervention or baseline to follow-up) did not significantly differ between groups (*p* > 0.32). Similarly, the time elapsed between the last attended group session and the post-intervention or follow-up assessments showed no significant difference between the groups (both *p* > .21) (see Supplementary Table S2).

### Feasibility

The feasibility of the study was assessed through various markers to evaluate the practicality and acceptability of the MEDITAGING program. Detailed information about these markers can be found in Table 2.

**Table 2:** Feasibility markers of the study based on Mace et al. (2023).

Marker	Criteria
Enrollment in the program	89 participants out of 151 (85.9 %) of screened participants enrolled in the study.
Program acceptability	63 out of 89 participants (70.8 %) attended >3 sessions.
Credibility expectancy	45 out of 56 participants (80.4 %) scored above the midpoint of the credibility scale.
Program fidelity	The clinician completed all proposed tasks.
Homework adherence	The majority of participants did not complete the diary for their home practices; therefore, homework adherence could not be reliably assessed. Several participants reported difficulties with written records, likely related to low literacy and unfamiliarity with the concept of homework assignments.
Program safety and adverse events	No participant reported adverse effects.

Regarding program acceptability, a substantial proportion of participants attended the first session (68.6 %), demonstrating their willingness to engage in the program from the outset. Moreover, a significant percentage of participants (70.8 %) continued to attend more than three sessions, indicating a consistent level of participation among those who joined the study. On average, participants took part in 6.38 sessions out of 8 sessions. The frequency of class attendance was comparable between the groups (*M*_HPP_ = 4.09 ± 3.09; *M*_MBSR_ = 5.14 ± 2.92; *t* (87) = - 1.64, *p* = .41). The majority of dropouts (*n* = 13) occurred before the first session, indicating that they were not influenced by the intervention itself. The primary reasons for dropout included COVID-19 infection (*n* = 2), interpersonal conflicts with another group member (*n* = 3), travel commitments (*n* = 3), health issues (*n* = 3), or unspecified reasons (*n* = 4). No participant reported difficulties adhering to the program due to financial constraints. However, some employed participants mentioned challenges balancing the practice with their work commitments.

### Participant compliance with the interventions

As shown in Supplementary Fig. S1, approximately 69 % of the total participants attended the first group session, with attendance gradually decreasing over subsequent sessions, reaching around 37 % of attendance by the end. On average, participants attended 51 % of the structured sessions. However, considering participants included in the modified intention-to-treat analysis, the average attendance was 70.7 %.

Participants from both groups perceived the intervention as credible, with 80 % of participants scoring above the midpoint in the Credibility/Expectancy Questionnaire, indicating a high level of trust in the program’s effectiveness (see Table 2).

### Analysis of primary and secondary outcomes

**Fig. 3:**
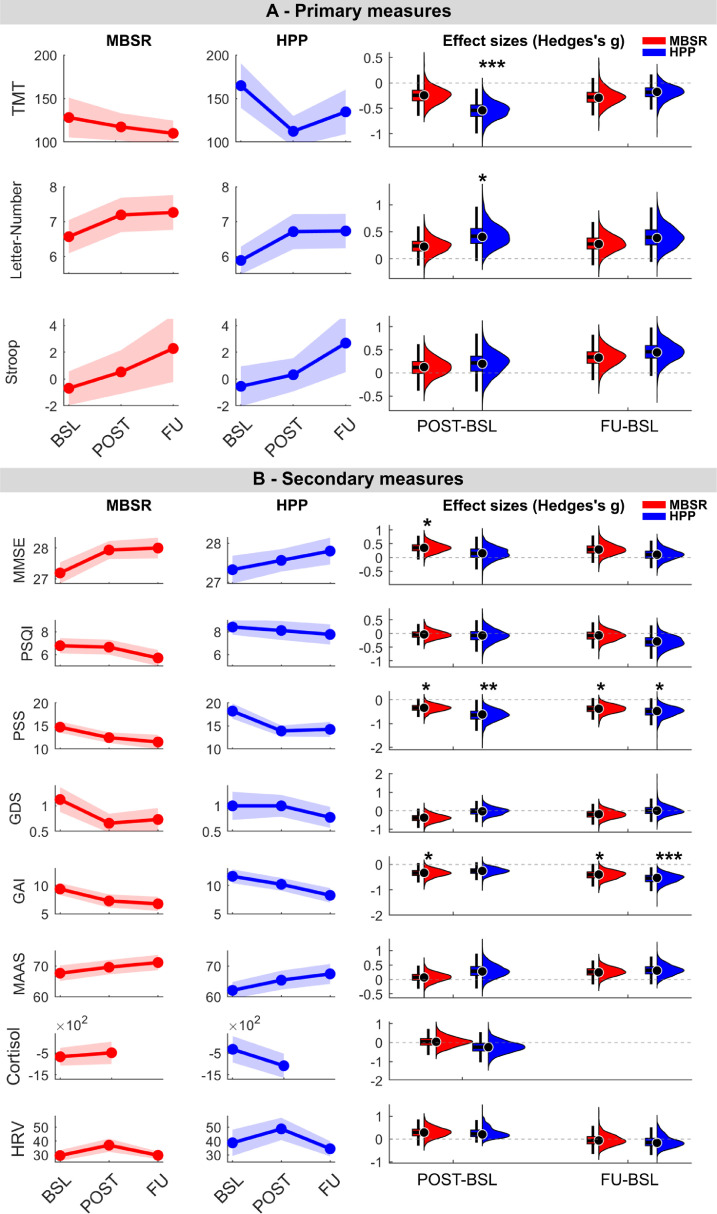
Primary (A) and secondary (B) outcomes across assessment sessions. Effect size distributions (C).

In the linear mixed-effect model analysis, no significant group x time interaction was observed for either primary or secondary outcomes (see Table 3). However, a significant main effect of time was found for the primary outcome, the Letter-Number Sequencing score (*β* = 0.46, *SE* = 0.23, *p* = 0.04, 95 % CI [0.02, 0.90]; Fig. 3A and Supplementary Fig. S2), indicating an overall improvement across groups. A sensitivity analysis, controlling for age, sex, education, and number of sessions attended, did not alter the direction of the results, with only subtle changes observed (see Table S3 for detailed results). Both groups exhibited increased Letter-Number Sequencing scores at post-intervention (*β* = 0.72, *SE* = 0.29, *p* = .03, 95 % CI [0.04, 1.40]) and follow-up (*β* = 0.79, *SE* = 0.30, *p* = .03, 95 % CI [0.08, 1.50]) compared to baseline. Bootstrap analysis of the effect sizes (Fig. 3A, on the right) confirmed significant improvement in the performance for the Letter-Number Sequencing (*g* = 0.40, 95 % [CI 0.06, 0.87]) but only for the HPP group. Additionally, the bootstrap analysis of the effect sizes for the TMT revealed a significant effect for the HPP group at post-intervention compared to baseline (*g* = - 0.54, 95 % *CI* [−0.90, - 0.24]), even though this effect was not detected in the prior LMM analysis. These findings are visually represented in Fig. 3A, which suggests an increase in Letter-Number Sequencing and a decrease in TMT scores at post-intervention assessments. Overall, these findings offer preliminary insights into the potential impact of both interventions on improving executive function.

**Table 3:** Modified intention-to-treat means and standard deviations for primary and secondary outcome measures.

	BSL	POST	FU	*Model estimate (SE) and p*-value		
				Group comparison	Time comparison	Group x Time
Sample size						
*HPP* * MBSR*	*n* = 26 *n* = 32	*n* = 26 *n* = 32	*n* = 22 *n* = 26	–	–	–
Letter-Number						
* HPP* * MBSR*	5.88 (1.99) 6.56 (2.71)	6.71 (2.48) 7.19 (2.80)	7.26 (2.60) 6.73 (2.31)	*0.65 (0.65)* *p* = .32	**0.46**⁎**(0.23)** ***p* = .04**	*−0.*10 (0.30) *p* = .74
STROOP						
* HPP* * MBSR*	−0.55 (7.61) −0.69 (7.03)	−0.32 (6.21) −0.53 (8.78)	2.69 (9.71) 2.28 (12.75)	−0.15 (2.24) *p =* .95	1.64 (1.00) *p* = .11	−0.11 (1.34) *p* = .94
TMT						
* HPP* * MBSR*	164.75 (125.77) 128.10 (123.41)	112.26 (84.72) 117.31 (84.59)	134.63 (111.43) 109.96 (76.11)	−21.44 (27.01) *p* = .43	−14.98 (9.83) *p* = .13	1.78 (12.99) *p* = .89
MMSE						
* HPP* * MBSR*	27.32 (1.80) 27.19 (1.96)	27.56 (1.45) 27.94 (1.63)	27.80 (1.54) 28.00 (1.72)	−0.08 (0.44) *p* = .85	0.11 (0.18) *p* = .55	0.23 (0.25) *p* = .35
PSQI						
* HPP* * MBSR*	8.42 (3.48) 6.78 (3.77)	8.12 (4.17) 6.66 (3.72)	7.77 (4.15) 5.70 (3.67)	−1.62 (0.98) *p* = .10	−0.39 (0.37) *p* = .29	0.13 (0.49) *p* = .80
GDS						
* HPP* * MBSR*	1.00 (1.41) 1.13 (1.39)	1.00 (1.08) 0.66 (1.07)	0.77 (0.97) 0.73 (1.15)	0.01 (0.30) *p* = .99	−0.08 (0.13) *p* = .53	−0.09 (0.17) *p* = .62
GAI						
* HPP* * MBSR*	11.62 (6.20) 9.38 (5.86)	10.20 (5.62) 7.28 (6.51)	8.27 (5.96) 6.78 (6.50)	−2.61 (1.58) *p* = .10	**−1.70**⁎⁎⁎**(0.47)** ***p* < .001**	0.56 (0.63) *p* = .38
PSS						
* HPP* * MBSR*	18.20 (8.28) 14.71 (6.76)	13.88 (6.01) 12.42 (6.40)	14.23 (7.67) 11.48 (8.10)	−3.11 (1.85) *p* = .09	**−2.17**⁎⁎**(0.71)** ***p* < .01**	0.73 (0.95) *p* = .44
MAAS						
* HPP* * MBSR*	62.08 (13.60) 67.68 (14.10)	65.40 (15.48) 69.65 (13.11)	67.45 (15.29) 71.15 (12.72)	5.17 (3.60) *p* = .15	2.58 (1.35) *p* = .06	−0.85 (1.82) *p* = .64
Cortisol						
* HPP* * MBSR*	−326.59 (2851.72) −669.63 (2291.43)	−1085.52 (2614.48) −486.44 (2687.51)	–	−341.64 (722.96) *p* = .64	−755.19 (744.63) *p* = .32	928.50 (986.93) *p* = .35
HRV						
* HPP* * MBSR*	38.68 (47.94) 29.54 (21.67)	48.68 (4.13) 36.95 (26.43)	34.44 (24.33) 29.73 (13.73)	−10.55 (7.82) *p* = .18	−1.46 (3.55) *p* = .68	1.69 (4.75) *p* = .72

Continuous variables are presented as *M* (*SD*), categorical variables as frequency (%).

Abbreviations: BSL, Baseline session; POST, Post-intervention session; FU, Follow-up session; HPP, Health Promotion Program; HRV, Heart Rate Variability; MAAS, Mindfulness Attention and Awareness Scale; MBSR, Mindfulness-Based Stress Reduction; MMSE, Mini-Mental State Examination; PSQI, Pittsburgh Sleep Quality Index; GDS, Geriatric Depression 5 Item Scale; GAI, Geriatric Anxiety Inventory; PSS, Perceived Stress Score; STROOP, Stroop color-word; TMT, Trail Making Test.

⁎ *p* < .05.

⁎⁎ *p* < .01.

⁎⁎⁎ *p* < .001.

Graphs A e B depict changes in *M* scores across assessment sessions (Baseline, BSL; Post-Intervention, POST; Follow-Up, FU) for the Health Promotion Program (HPP, shown in blue) and the Mindfulness-Based Stress Reduction (MBSR, shown in red). For Letter-Number Sequencing and Mini-Mental State Examination (MMSE), higher composite scores indicate better performance whereas for the Trail Making Test (TMT), Stroop Color-Word, Pittsburgh Sleep Quality Index (PSQI), Perceived Stress Score (PSS), Geriatric Depression 5 Item Scale (GDS), Geriatric Anxiety Inventory (GAI), Mindfulness Attention and Awareness Scale (MAAS), higher scores indicate worse performance. Shading area represents the standard error of the mean. In Graph C, the violin plots illustrate the distribution of effect size changes derived from 1000 bootstrap samples. The filled black circle represents the average of the distribution. *p < .05; **p < .01.

Regarding secondary outcomes, visual inspection of Fig. 3B suggests that both interventions may have contributed to general improvements across nearly all secondary outcomes. These patterns were broadly consistent with the statistical results, which showed significant main effects of time for measures of anxiety and stress. Specifically, a main effect of time was also observed in GAI (*β* = - 1.70, *SE* = 0.47, *p* < .01, 95 % CI [- 2.62, - 0.78]), with lower scores at post-intervention (*β* = - 1.86, *SE* = 0.60, *p* < .001, 95 % CI [- 3.28, - 0.43]) and follow-up (*β* = - 2.74, *SE* = 0.63, *p* < .001, 95 % CI [- 4.25, - 1.24]) relative to baseline. Similarly, a main effect of time was also observed for the Perceived Stress Scale (PSS) scores (*β* = - 2.17, *SE* = 0.71, *p* < .01, 95 % CI [- 3.55, - 0.78]), with reductions at post-intervention (*β* = - 3.21, *SE* = 0.89, *p* < .01, 95 % CI [- 5.33, - 1.09]) and follow-up (*β* = −3.43, *SE* = 0.94, *p* < .01, 95 % CI [- 5.66, - 1.21]) relative to baseline. These results suggest that both interventions decreased anxiety and stress levels.

Consistent with these findings, bootstrap analyses of the effect sizes suggest that the MBSR demonstrated lower GAI scores at post-intervention (*g* = - 0.33, 95 % CI [- 0.65, - 0.08]) and follow-up (*g* = - 0.40, 95 % CI [- 0.75, - 0.11]) than at baseline, while the HPP group showed significant effect sizes at follow-up compared to the baseline (*g* = - 0.53, 95 % CI [- 0.95, - 0.25]). Additionally, reductions in PSS scores for both groups at post-intervention (MBSR: *g* = - 0.33, 95 % CI [- 0.64, - 0.05]; HPP: *g* = - 0.61, 95 % CI [- 1.20, - 0.20]) and follow-up (MBSR: *g* = - 0.37, 95 % CI [- 0.75, - 0.06]; HPP: *g* = - 0.47, 95 % CI [- 1.00, - 0.09]) relative to baseline were observed in the bootstrap analysis. Although no significant effect was found in the main statistical analysis of MMSE, the bootstrap analysis indicated higher scores at post-intervention compared to the baseline for the MBSR group (*g* = 0.35, 95 % CI [0.04, 0.71]). A complete table with the effect sizes and respective confidence intervals can be found in Supplementary Material Table S4.

Additionally, to verify the robustness of the results and to assess the impact of including participants with lower attendance, sensitivity analyses were conducted on a subsample of participants who completed 60 % or more of the sessions (see Supplementary Table S6). The findings from this subset were consistent with the overall conclusions drawn from the full sample, which included participants attending at least three out of nine sessions (33 %).In sum, our findings suggest that participants experienced improvements in both executive and affective functioning following the group interventions, irrespective of the intervention type. These improvements persisted at the follow-up, conducted around one to three months after the intervention.

### Exploratory correlation analyses

Further exploratory analyses were conducted to evaluate the correlation between improvements in the Letter-Number Sequencing (in the analysis above identified as the primary outcome demonstrating a significant time effect) and participant’s characteristics. These characteristics included age, years of education, sex, and number of completed sessions, as well as baseline scores of self-reported and physiological measures. Improvements were calculated as the difference between post-intervention and baseline. Our correlation analysis revealed no significant findings for any analysis (all *p* values > 0.09, Supplementary Fig. S3). Additionally, although the percentage of men (24 %) across the two conditions in the post-intervention assessment was smaller than women, Mann-Whitney *U test* confirmed that improvements in Letter-Number did not differ significantly between men and women (*z* = 0.087, *p* = .93; Supplementary Fig. S4). Our results suggest that improvements in executive function following group interventions are not related to individual characteristics.

Furthermore, since the follow-up assessments were not conducted on the same day for all participants, the interval between the post-intervention assessment and follow-up varied among them. To investigate whether this variability in the number of days between assessments had any impact on the observed outcome scores, a regression analysis was performed on the outcomes where significant effects were identified in the previous analysis (i.e., Letter-Number Sequencing, GAI, and PSS). The results were not significant (all *p* > .11, see Supplementary Fig. S5), indicating that the timing between assessments did not significantly influence the outcomes.

### Adverse effects

No adverse effects were reported by any of the participants, indicating the overall safety of the MEDITAGING interventions. Additionally, the linear mixed models did not show significant difference between the groups in the PANAS Positive Affect and VAS (Visual Analogue Scale) questionnaires. However, for PANAS Negative Affect score, the interaction between group and time was significant (*β* = −0.78, *SE* = 0.38, *p* = .04). Post-hoc analysis identified a marginally significant reduction in negative PANAS score over time in the MBSR group, specifically between session 2 and session 6 (*β* = −5.42, *SE* = 1.93, *p* = .08; Supplementary Fig. S6 and Supplementary Table S5). These results suggest that the interventions are safe and comparable, with potential for MBSR to decrease negative emotions.

## Discussion

In the MEDITAGING trial, we explored the impact of the MBSR, compared to a HPP, on cognitive, affective, and physiological measures among older Portuguese-speaking immigrants primarily from low socioeconomic backgrounds in Luxembourg. Our preliminary findings suggest that both group programs are feasible and well-accepted among the targeted population, with no adverse effects reported. Participants showed improvements in executive functioning, perceived stress, and anxiety symptoms following the group interventions, irrespective of the intervention type. These improvements were sustained at the follow-up session, which occurred approximately one to three months after the interventions. The most robust results were found in the self-reported measures of anxiety and stress, which were secondary outcomes. The analysis of these secondary measures showed higher and significant effect sizes in both groups, ranging from |0.33| to |0.61|, indicating moderate to large effects. The significance of these findings is underscored by research that consistently links constructs such as working memory, stress, and anxiety with healthy aging (Hernandez et al., 2014; Luo et al., 2022; Memel et al., 2019). Effect size analysis also suggested improvements in the MMSE, a measure of general cognitive function, at the post-intervention in comparison to the baseline, for the MBSR group. Overall, our results did not reveal an evident superiority of mindfulness over a HPP in enhancing executive functioning, general cognition, physiological, or self-reported outcomes. Instead, both interventions seem to have a positive impact on cognitive and affective measures.

Due to the nature of the active control condition, we anticipated benefits for both intervention groups. Nevertheless, we hypothesized that the MBSR group would exhibit greater enhancements in executive functioning, as attention is one of the primary targets of the program (Lutz et al., 2008; Verhaeghen, 2021). Additionally, we expected that the MBSR group would experience significant stress relief, especially evidenced by lower cortisol levels (Wetherell et al., 2017) and increased heart rate variability (Nijjar et al., 2014). However, consistent with a previous study by Lenze et al. (2022), our findings did not confirm these hypotheses. We did not observe significant differences between the MBSR and the HPP in executive functioning or in secondary measures, such as the PSQI, which assesses sleep quality, aligning with findings by Perini et al. (2023).

This lack of difference in the effects of the two interventions is in line with existing literature, which shows that significant differences are often not observed when using an active control group for comparison (Lannon-Boran et al., 2024; Whitfield et al., 2022). In our study, we incorporated an active control condition to address ethical and methodological challenges. This involved delivering a structured Health Promotion Program to the control group. This enabled both groups to benefit from the interventions, as both conditions were well-structured, group-based lifestyle interventions. Participants in the active control condition reported improved social connectivity and engagement. This was evidenced by participants forming social networks and reporting connections outside the sessions, a phenomenon less observed in the MBSR group, whose activities were more introspective in nature. The active control sessions, which included interactive activities, fostered a greater sense of social cohesion and support among participants. This social interaction, along with the health literacy taught in the sessions, could have played a crucial role in enhancing overall self-care and well-being and may explain some of the observed benefits in the active control group. This is particularly significant, as many participants reported feelings of isolation and limited financial, psychological, and coping resources during the initial baseline interviews. Thus, this study underscores the value of incorporating social elements and practical health education into interventions in older adults from vulnerable populations.

It is also worth noting that nonpharmacological interventions, including mind-body exercises, meditation, non-invasive brain stimulation, and physical exercises, have been suggested to enhance cognition compared to passive controls, with no discernible differences in efficacy across these modalities (Seok et al., 2024). Although the exact mechanisms remain unclear, the pathways likely include cognitive enhancement, increased brain plasticity, and stress reduction, targeting several bio-behavioral mechanisms shared across various interventions (Ninot, 2021). This suggests that the improvements observed in both the MBSR and HPP groups could be attributed to these underlying mechanisms, further validating the effectiveness of structured, group-based interventions in enhancing cognitive and affective outcomes. This observation indicates that various nonpharmacological interventions can yield benefits, regardless of the specific modality, also meaning that there is a great potential to tailor interventions considering individual needs and preferences.

Previous meta-analysis has shown that mindfulness is superior to passive control comparisons, such as wait-list, in reducing distress (Galante et al., 2023; Whitfield et al., 2022). Therefore, the improvements observed in our trial are unlikely due to test-retest effects and may instead reflect genuine improvements in both groups. Although including a negative control condition could have strengthened this argument by attributing changes specifically to the interventions and differentiating them from natural improvements or test-retest effects. We believe several factors mitigate this concern. Firstly, expectancy effects have been shown not to mediate the outcomes of mindfulness interventions (Haddad et al., 2020). Secondly, the interval of at least two months between baseline and post-intervention assessments was likely sufficient to allow a potential washout effect, reducing the likelihood that the improvements were solely due to repeated testing (Lemay et al., 2004). These aspects strengthen the argument that the cognitive improvements observed were not merely due to participants becoming more familiar with the test format. Additionally, the self-reported questionnaires used in our study are designed to assess state rather than trait characteristics, making them more susceptible to changes over short periods.

Although prior research has suggested that mindfulness can lead to positive changes in physiological measures such as reductions in morning cortisol levels and changes in parameters of HRV (Brand et al., 2012; Nijjar et al., 2014), no significant effects of the interventions were verified in our physiological measures. A recent study with other biological markers such as inflammatory measures (i.e., interleukin 6 or C-reactive protein) found MBSR effects compared to a health enhancement control in proinflammatory gene regulation (Lindsay et al., 2024), which might be a more promisor biological outcome for future trials.

Exploratory analyses also revealed that the improvements observed in executive functioning for both groups were not correlated with individual characteristics such as baseline scores on self-reported questionnaires, physiological measures, age, sex, education, and the number of attended sessions. This suggests that the benefits of the interventions were broadly applicable across different subsets of our study population, regardless of their initial emotional or physiological status. The absence of significant correlations might indicate that the interventions offer generalized benefits rather than being effective only for specific baseline characteristics. Nonetheless, this warrants deeper investigation. Future research, particularly on mindfulness, could focus on the effects of socio and intellectual capital on mindfulness outcomes (Bourdieu, 2018). It is important to note that the study may have lacked statistical power, especially to detect additional exploratory associations, as the sample size calculation was designed for the primary analysis and based on a population with higher education levels, which may not fully represent our target population (Moynihan et al., 2013). Additionally, the high dropout rates have further limited the power of the analyses.

In terms of session adherence, on average, participants included in the analysis attended 70.7 % of the MEDITAGING sessions. It is also important to note that in 31.5 % of enrolled participants did not attend the first session in our study, which is similar to a significant study on cognitive training, in which 37 % of participants did not attend even a single activity of the program (Turunen et al., 2019). Additionally, similar to our findings, where 37 % of participants attended the last group session and 65 % participated in at least one of the post-assessment sessions, a mindfulness-based study with Portuguese-speaking immigrants in the USA also observed a decline in participant attendance over the course of the sessions, with only 30 % attending the final session and 62.5 % completing the post-intervention assessment (Trombka et al., 2021).

We postulated that higher adherence could potentially result in more significant positive effects. However, we failed to find a correlation between number of attended sessions and neuropsychological improvements. Additional sensitivity analyses presented at Supplementary Table S6 did not indicate greater effects compared to the main findings for the total participant group. Furthermore, an earlier study that followed participants for an extended duration with boost sessions did not find significant effects in comparison to active controls (Lenze et al., 2022).

Regarding the limitations of this study, several factors should be considered when interpreting our findings, including dropout rate, homework adherence and recording, and outcome measures. First, our study faced a 34.8 % dropout rate, primarily due to the challenges encountered by older immigrants, such as traveling back to their home countries, work or caregiving responsibilities, bereavement, and high number of sick days mainly related to the pandemic or chronic pain. These values are consistent with findings in the literature, which report attrition rates ranging from 0 to 64 % (*M* = 23 %) in mindfulness-based interventions for older populations (Geiger et al., 2016). Similarly, in clinical samples, dropout rates have also shown wide variability, ranging from 5 % to 63 %, with an average attrition rate of 29 % (Nam & Toneatto, 2016). Additionally, a previous review focusing on a population of self-identified Latino or Hispanic participants showed that attrition rates can reach as high as 75 % (Cotter & Jones, 2020). These findings highlight the typical challenges of maintaining high adherence in such studies. It is important to account for low adherence rates when calculating sample size for future studies to ensure that sufficient power is maintained despite potential dropouts.

That said, we do not believe participants were uninterested in the program activities. This is supported by the fact that 80 % rated the program above the midpoint on the credibility scale, as well as by the subjective benefits they reported, such as increased ability to cope with stress, breaking free from automatic behaviors, and enhanced social connection. Many participants also expressed a desire for the program to continue after the study ended, with some even coordinating with senior clubs to organize similar activities. Nonetheless, participation came at a cost in terms of time and effort (Nam & Toneatto, 2016). Many participants have spent their lives in caregiving and work roles and are not accustomed to prioritizing their own needs. As a result, other life responsibilities often took precedence. Additionally, participants may not have developed the routines of attending classes or completing homework tasks that are more typical in formal education settings. As one participant stated: "*We’re not used to having to do that, and we think it’s like we have to make an effort to do the homework* a*nd I think that many people in the group who dropped out did so because of this […] There are days when, for example, I didn’t feel like writing. […] But that’s what bothered me the most. The homework tasks because I don’t really like writing. But other than that, I think everything was good*”.

Moreover, this challenge with the homework was also reflected in our second main limitation: the inability to systematically analyze the homework tasks. Participants often did not return the activity sheets. Many expressed difficulties in filling out the forms and some returned blank pages. When asked whether this meant they had not completed the tasks, several clarified that they had in fact done them but did not know how to properly fill in the sheet. This limitation is particularly important given that home practice is a core element of mindfulness-based interventions, being associated with its positive outcome (Parsons et al., 2017). Without clear evidence of whether participants engaged with the homework, it is difficult to assess whether this could be a contributing factor to the lack of significant effects compared to the HPP group.

Building on these insights, several strategies can be considered to enhance participant engagement and adherence in class and home activities. Regarding session attendance, these include:

1. Reducing session duration while increase session frequency: To reduce dropout, it may be beneficial to shorten the length of the sessions and reduce the interval between them. For example, instead of weekly sessions of 2.5-h as in our study, each session could be split into two shorter segments, delivered twice a week. This could facilitate a smoother rhythm for participants to follow. Additionally, this structure might make it easier for participants to find one hour in their schedules, rather than committing to longer sessions that could feel more demanding.
2. Co-creating the agenda with participants: Prioritizing the participants’ needs and schedules is crucial for improving engagement and adherence. Many older migrants balance multiple responsibilities, such as caregiving or work, which can make it difficult to attend sessions or complete homework tasks. It may be helpful to involve participants in co-creating the agenda. By discussing and determining the most suitable days and times for sessions together, we can better accommodate their schedules and reduce the strain of participating. They could also be actively involved in the planning the program and home activities can be better adapted to their individual circumstances. For instance, adjusting the content to focus on participants' priorities.
3. Check-in calls for ongoing support: regular phone calls could be implemented to provide consistent follow-up and address any challenges participants might be facing (e.g., Trombka et al., 2021). These calls could also serve to monitor their progress with home practice, offer tailored support, and promote a sense of commitment.
4. Follow-up for session absences: If a participant misses a session, it is important to reach out to offer support and check in. This proactive approach not only shows participants that their involvement is valued, but it also provides an opportunity to address any issues at an early stage, ensuring they feel supported throughout the program.
5. Activity monitoring during sessions: It is essential to regularly evaluate each activity during the group sessions. This can be done by implementing quick, easy, and anonymous feedback methods at the end of each activity. Rather than relying on written forms, a simple, non-verbal approach, such as using traffic signal icons or emoji-based responses, can be used to instantly capture participants' reactions right after each activity.
6. Integration with healthcare services: Integrating the program into healthcare services could increase participant commitment and help ensure that the program is viewed as an extension of their regular medical care (e.g., Trombka et al., 2021).
7. Providing additional resources for social connection:
  While the facilitator guided discussions in alignment with the session’s theme and objectives, it became clear that providing additional space for informal interaction would be beneficial.
8. Separate orientation group session: Another option could be to hold a separate orientation session after to the baseline assessment. This session would focus on ensuring that participants fully understand the purpose and structure of the group activities, as well as the importance of their commitment and addressany barriers to initial engagement in the intervention. Also, this could help setting realistic expectations from the beginning.
9. Hybrid program option: A potential solution could be offering a hybrid program that allows individuals to participate remotely. While in-person interactions are likely more beneficial, offering remote participation could increase adherence to the program. However, previous studies on online interventions have also shown high dropout rates, often with almost one-third-of participants not completing the program (Brodaty et al., 2025).

Regarding home activities, the following suggestions are recommended:

(1) Simplify the design of homework materials by providing instructions in audio and video format and using a workbook with simpler language.
(2) Consider alternative formats for recording practice with the aid of equipment such as recorders or apps, to accommodate participants with varying levels of literacy and comfort with written tasks. Alternatively, a diary format with checkboxes could be used.
(3) Reduce the time commitment or offer smaller, more manageable task alternatives, ensuring the home tasks feel less overwhelming.

Final limitations concern the measures used. Further research is needed to support a more thorough psychometric validation of the instruments among low-educated older migrant populations. Additionally, it would be valuable to develop guidelines for studies of this nature, enabling more consistent evaluation and allowing for comparisons across different studies. Participant dropout resulted in a smaller final sample than originally planned, potentially affecting the statistical power of our analyses. In addition, the inclusion of multiple primary outcomes may have increased the risk of Type I errors, as testing several outcomes can raise the likelihood of false-positive findings. However, this approach was intentionally chosen due to the limited existing evidence regarding the cognitive effects of these intervention in this specific population. Given the multidimensional nature of executive functioning (Miyake et al., 2000), examining a broad range of cognitive domains would allow us to identify which one may be most responsive to intervention, thereby informing outcome selection in future research. Our findings provide preliminary insights that can help guide future studies in narrowing the focus to a single primary outcome or constructing a well-defined composite score.

One strength of this study was that we offered the intervention in the participants' native language, which we believe was crucial for its acceptance and effectiveness. However, future studies might consider designing or validate specific mindfulness sessions for this population, as we observed that many participants had difficulties in understanding the mindfulness vocabulary, achieving profound self-insights, and working with reading and writing materials. While we made small adjustments to the program to meet the needs of participants, studies specifically focused on tailoring the sessions for this group could potentially maximize their efficacy. Additionally, it could be valuable to quantitatively control the depth of the meditation practice (Piron, 2022).To the best of our knowledge, this study is the first of its kind conducted in this demographic in Europe. Addressing the cognitive and mental health needs of older immigrants is critical due to their heightened vulnerability to cognitive decline, exacerbated socioeconomic disadvantages, and the challenges associated with immigration and integration. Given their increased risk of dementia (Parlevliet et al., 2016), preventive measures for this population are imperative, especially considering that aging research has historically focused on well-educated individuals (e.g., Lannon-Boran et al., 2024). Therefore, our findings offer valuable insights into the importance of delivering these types of structured group interventions to vulnerable populations, such as older immigrants. Importantly, these activities are low-cost and easy to implement, which reinforces their significance and enhance their sustainability.

## Conclusion

While our preliminary findings did not show significant differences between MBSR and HPP interventions, the improvements observed in executive function, perceived stress, and anxiety following both interventions suggest that structured, group-based lifestyle interventions may offer benefits for affective states and cognition in vulnerable older adults. The findings highlight the need for further research to explore tailored approaches that could enhance engagement and effectiveness in this population. Our study emphasizes the role of these interventions as potential strategies within the realm of healthy aging and dementia prevention.

## Data availability

Datasets may be available under reasonable request.

## Ethical standards

The authors assert that all procedures contributing to this work comply with the ethical standards of the relevant national and institutional committees on human experimentation and with the Helsinki Declaration of 1975, as revised in 2008.

## Funding

This work was entirely supported by the Luxembourg National Research Fund (FNR) [grant numbers 15240063 and C23/SC/17995805]. AS-F is supported by the Foundation for Science and Technology (FCT) and the Portuguese Ministry of Science, Technology and Higher Education, through the national funds, within the scope of the Transitory Disposition of the Decree No. 57/2016, 29th of August, amended by Law No. 57/2017 of 19 July and her work is conducted at the Psychology Research Centre (CIPsi), School of Psychology, University of Minho, supported by the Foundation for Science and Technology (FCT) through the Portuguese State Budget [Ref.: UIDB/PSI/01662/2020]. The study funder has no role in any stage of this study.

## CRediT authorship contribution statement

**Ana C. Teixeira-Santos:** Conceptualization, Methodology, Formal analysis, Investigation, Data curation, Writing – original draft, Visualization, Project administration, Funding acquisition. **Leandro Gomes:** Methodology, Data curation, Writing – review & editing. **Diana R․ Pereira:** Investigation, Writing – review & editing. **Fabiana Ribeiro:** Methodology, Investigation, Writing – review & editing. **Joana Carvalheiro:** Formal analysis, Validation, Writing – review & editing, Visualization. **Catarina Godinho:** Investigation. **Anabela Silva-Fernandes:** Methodology. **Etienne Le Bihan:** Visualization, Validation, Writing – review & editing. **Carine Federspiel:** Resources, Funding acquisition. **Jean-Paul Steinmetz:** Resources, Supervision, Funding acquisition, Methodology, Writing – review & editing. **Anja K․ Leist:** Resources, Methodology, Supervision, Funding acquisition, Writing – review & editing.

## Declaration of competing interest

The authors declare that they have no known competing financial interests or personal relationships that could have appeared to influence the work reported in this paper.

## Appendix Supplementary materials

Image, application 1
