# Supplementary material for: A randomized clinical trial of mindfulness training versus a health promotion program: Impact on cognitive and mental health in older immigrants
Source: Int J Clin Health Psychol. 2025 Oct 24;25(4):100642. doi: 10.1016/j.ijchp.2025.100642 (PMC12593566; doi:10.1016/j.ijchp.2025.100642)
Supplement: Supplementary file 1 [file mmc1.pdf]

Supplementary material

A Randomized Clinical Trial of a Mindfulness Training versus a Health Promotion Program on Cognitive Function in Older Adults

HANDLING OF MISSING DATA

- Imputation was not performed. In the case of missing values for an outcome measure, the individual did not enter the statistical analysis of that outcome.
- For participants with a single measure of heart rate variability out of the two planned, we included only that specific value instead of calculating the mean of the two values.

PERCENTAGE OF CLASS ATTENDANCE

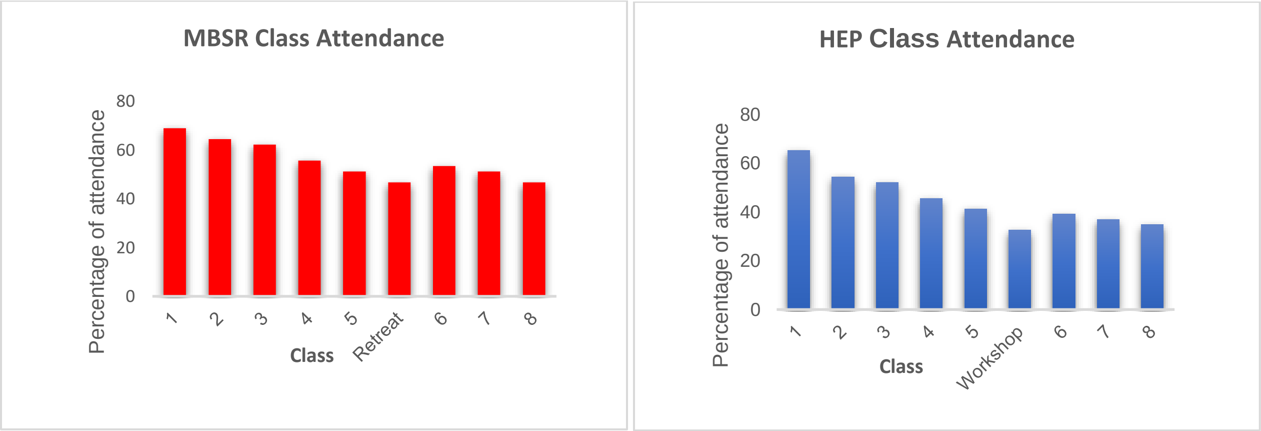

Figure S1. Percentage of class attendance averaged across participants in each of the groups*:* Mindfulness-Based Stress Reduction (MBSR, shown in red); Health Promotion Program (HPP, shown in blue).

BASELINE COMPARISON

Table S1

Characteristic Comparison Between Dropouts and Completers

	Age (years)		Education		MMSE		Sex (%)		Smokers		BMI	
	M	SD	M	SD	M	SD	Female	Male	Y	N	Mean	SD
Completers (n = 58)	62.66	6.37	7.53	3.81	27.28	1.87	44 (75.9%)	14 24.1%)	5 (8.6%)	53 (91.4%)	27.40	4.62
Dropouts (n = 31)	62.28	5.55	7.94	3.48	27.48	1.74	20 (64.5%)	11 (35.5%)	3 (9.7%)	25 (90.3%)	27.87	4.52
Group comparisons	*u*	*p*	*u*	*p*	*t*	*p*	χ^2	*p*	χ^2	*p*	*u*	*p*
	895.00	0.97	*814.*50	0.46	-0.49	0.63	1.29	.26	0.03	0.87	968.00	0.55

Abbreviations: BMI, Body Mass Index; MMSE, Mini-Mental State Examination.

Table S2

Comparison of Assessment Time Intervals Across Groups

	**HPP *M* (SD)**	**MBSR *M* (SD)**	***p***
Days between BSL and POST	84.92 (20.46)	88.28 (21.68)	.55
Days between BSL and FU	153.41 (31.78)	144.63 (28.81)	.32
Days between last attended group session and POST	14.47 (2.47)	13.26 (3.44)	.22
Days between last attended group session and FU	79.89 (5.77)	70.63 (5.15)	.22

Abbreviations: BSL, baseline session; FU, follow-up session; HPP, Health Promotion Program; MBSR, Mindfulness-Based Stress Reduction; POST, post-intervention session.

ASSESSMENT OF PRIMARY AND SECONDARY OUTCOMES WITH INDIVIDUAL TRAJECTORIES

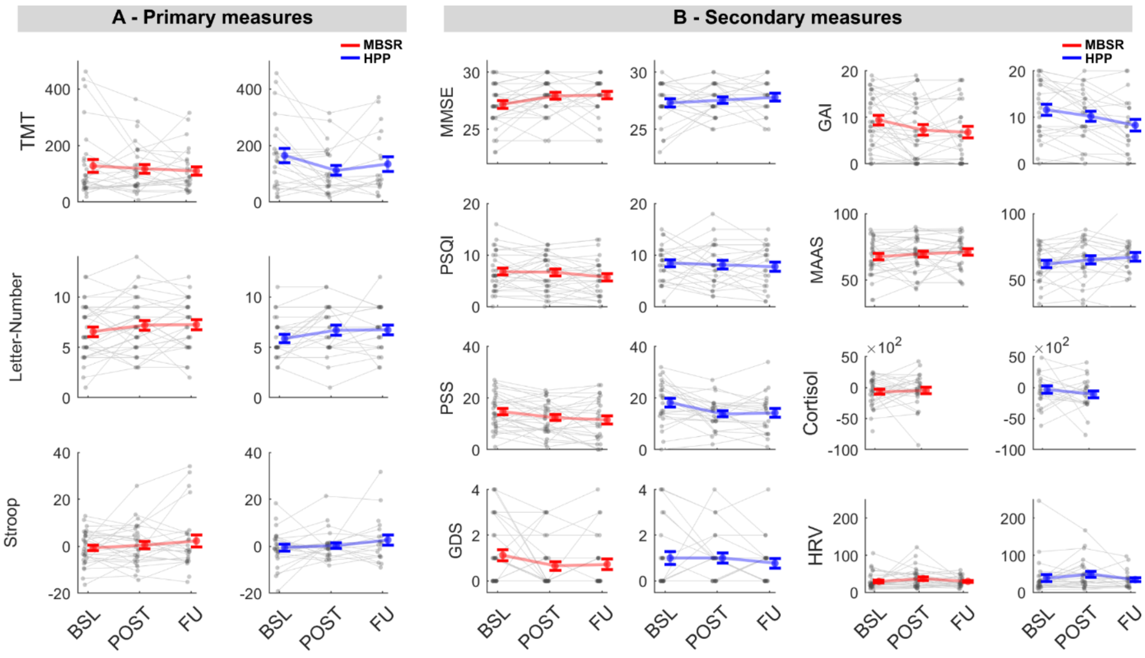

Figure S2.

Primary (A) and secondary (B) outcomes across the three assessment sessions with individual trajectories.

Graphs depict changes in mean scores across sessions (Baseline – BSL, Post-Intervention - POST, and Follow-Up - FU) in the Health Promotion Program (HPP, shown in blue) and the Mindfulness-Based Stress Reduction (MBSR, shown in red). Each subject's trajectory is represented by gray lines. More transparent (opaque) grey dots represent less (more) overlapping data points. Error bars represent the standard error of the mean.

Table S3

Sensitivity Analysis of the Main Model for Primary and Secondary Outcome Measures, Controlling for Age, Sex, Education, and Number of Sessions Attended

	***Model estimate and p*-value**		
	Group comparison	Time comparison	Group x Time
**Letter-number**			
	0.39 (0.57)	**0.48* (0.23)**	-*0*.13 (0.30)
	p = .49	***p* = .04**	*p* = .67
**STROOP**			
	-1.08 (2.27)	1.62 (1.00)	-0.07 (1.34)
	p = .64	*p* = .11	*p* = .96
**TMT**			
	-14.42 (24.00)	-15.41 (9.74)	1.56 (12.88)
	p = .55	*p* = .12	*p* = 0.90
**MMSE**			
	-0.24 (0.41)	0.10 (0.18)	0.22 (0.24)
	p = .55	*p* = .58	*p* = .36
**PSQI**			
	-1.54 (0.95)	-0.39 (0.37)	0.14 (0.49)
	p = .11	*p* = .29	*p* = .77
**GDS**			
	0.03 (0.31)	-0.08 (0.13)	-0.08 (0.17)
	p = .93	*p* = .51	*p* = .67
**GAI**			
	-2.07 (1.54)	**-1.70*** (0.47)**	0.58 (0.63)
	p = .18	***p* < .001**	*p* = .36
**PSS**			
	-2.19 (1.71)	**-2.17** (0.70)**	0.79 (0.95)
	p = .20	***p* < .01**	*p* = .41
**MAAS**			
	3.66 (3.58)	2.60 (1.35)	-0.91 (1.82)
	p = .31	*p* = .06	*p* = .62
**Cortisol**			
	-594.20 (705.03)	-794.95 (751.21)	889.41 (997.99)
	p = .40	*p* = .29	*p* = .38
**HRV**			
	-10.69 (8.13)	-1.5746 (3.55)	1.69 (4.75)
	p = .19	*p* = .68	*p* = .72

Abbreviations: MMSE, Mini-Mental State Examination; PSQI, Pittsburgh Sleep Quality Index; GDS, Geriatric Depression 5 Item Scale; GAI, Geriatric Anxiety Inventory; PSS, Perceived Stress Score, MAAS, Mindfulness Attention and Awareness Scale. *** $p < .001$, **$p < .01$; *$p < .05$.

Table S4

Effect size and Confidence Intervals for the Distribution of Effect Size Changes Derived From 1000 Bootstrap Samples.

	HPP				MBSR			
	Post-BSL		FU-BSL		Post-BSL		FU-BSL	
	g	95 %CI	*g*	95 %CI	*g*	95 %CI	*g*	95 %CI
TMT	**-0.54****	**[-0.90, -0.24]**	-0.18	[-0.50, 0.04]	-0.25	[-0.54, 0.05]	-0.29	[-0.57, 0.02]
Letter number	**0.40***	**[0.06, 0.87]**	0.38	[0.03, 0.80]	0.22	[-0.04, 0.51]	0.27	[-0.01, 0.60]
Stroop	0.20	[-0.26, 0.66]	0.44	[-0.03, 0.85]	0.13	[-0.25, 0.49]	0.33	[-0.09, 0.66]
MMSE	0.15	[-0.34, 0.57]	0.10	[-0.26, 0.50]	**0.35***	**[0.04, 0.71]**	0.29	[-0.08, 0.69]
PSQI	-0.07	[-0.57, 0.37]	-0.29	[-0.74, 0.12]	-0.03	[-0.30, 0.24]	-0.07	[-0.44, 0.29]
PSS	**-0.61****	**[-1.20, -0.20]**	**-0.47***	**[-1.00, -0.09]**	**-0.33***	**[-0.64, -0.05]**	**-0.37***	**[-0.75, -0.06]**
GDS	-0.03	[-0.43, 0.45]	0.00	[-0.46, 0.59]	-0.37	[-0.78, 0.00]	-0.19	[-0.66, 0.21]
GAI	-0.26	[-0.54, -0.02]	**-0.53*****	**[-0.95, -0.25]**	**-0.33***	**[-0.65, -0.08]**	**-0.40***	**[-0.75, -0.11]**
MAAS	0.26	[-0.13, 0.78]	0.31	[-0.01, 0.69]	0.07	[-0.23, 0.37]	0.24	[-0.04, 0.56]
HRV	0.21	[-0.06, 0.73]	-0.17	[-0.52, 0.46]	0.29	[-0.15, 0.70]	-0.06	[-0.46, 0.49]
Cortisol	-0.25	[-0.80, 0.35]	-	-	0.04	[-0.41, 0.62]	-	-

Abbreviations: BSL, Baseline; POST, Post-Training; FU, Follow-Up; HPP, Health Promotion Program; MBSR, Mindfulness-Based Stress Reduction. MMSE, Mini-Mental State Examination; PSQI, Pittsburgh Sleep Quality Index; GDS, Geriatric Depression 5 Item Scale; GAI, Geriatric Anxiety Inventory; PSS, Perceived Stress Score, MAAS, Mindfulness Attention and Awareness Scale; HRV, Heart Rate Variability. *p < .05; **p < .01; ***p < .001.

ADDITIONAL EXPLORATORY ANALYSES

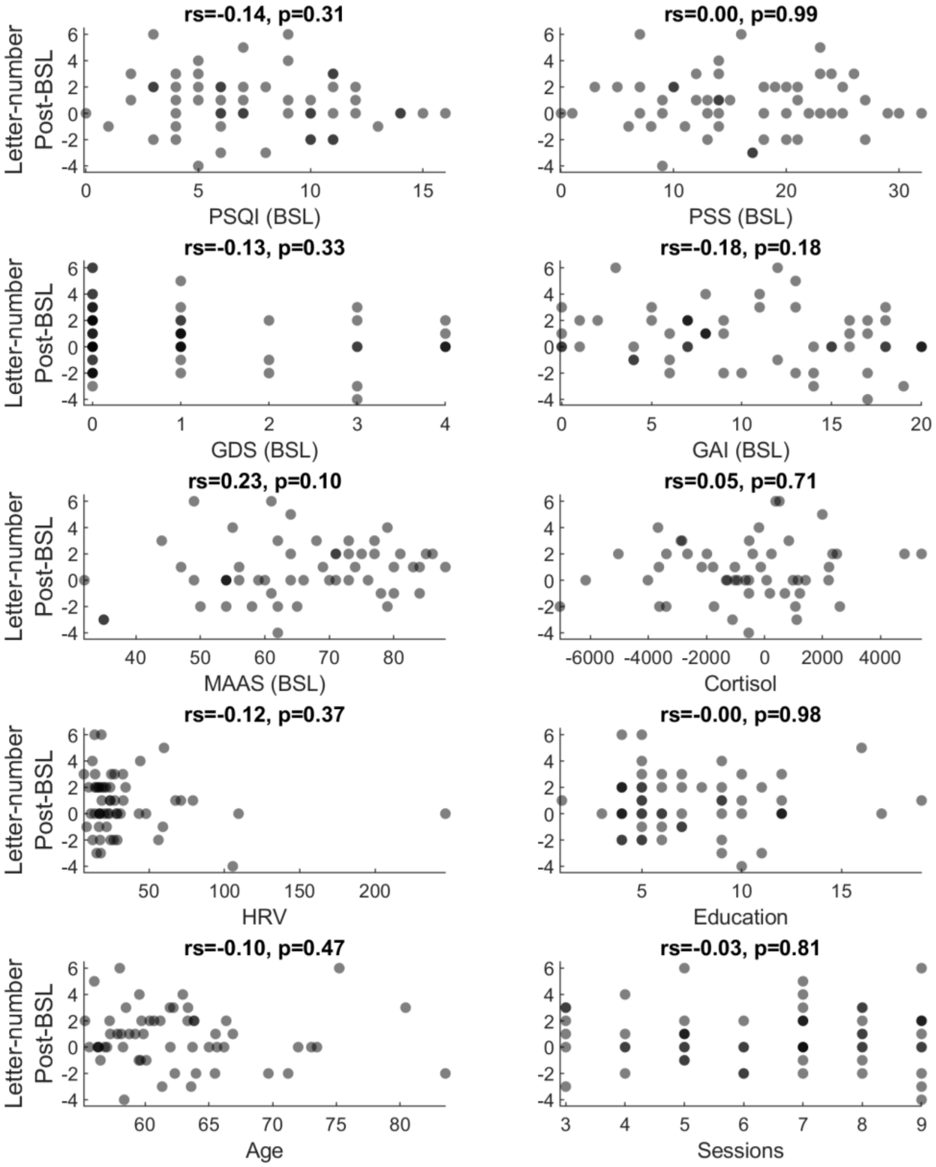

Figure S3.

Spearman correlation between gains (difference between scores in the baseline and post-intervention sessions) in the Letter-number sequencing and participant characteristics: age, years of education, number of completed sessions, and self-reported questionnaires. More transparent dots represent fewer overlapping data points, while more opaque dots indicate a higher number of overlapping data points. Both groups were merged in this analysis.

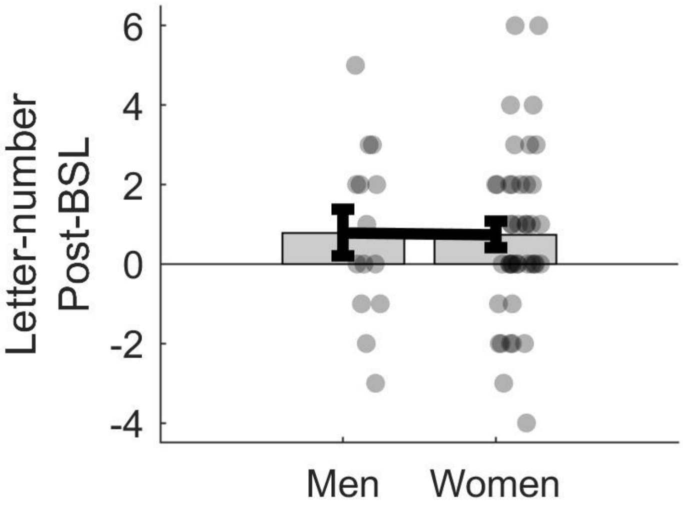

Figure S4.

Sex difference between Letter-Number Sequencing Scores between baseline and post-test sessions. More transparent dots represent fewer overlapping data points, while more opaque dots indicate a higher number of overlapping data points. Both groups were merged in this analysis. Error bars represent the standard error of the mean.

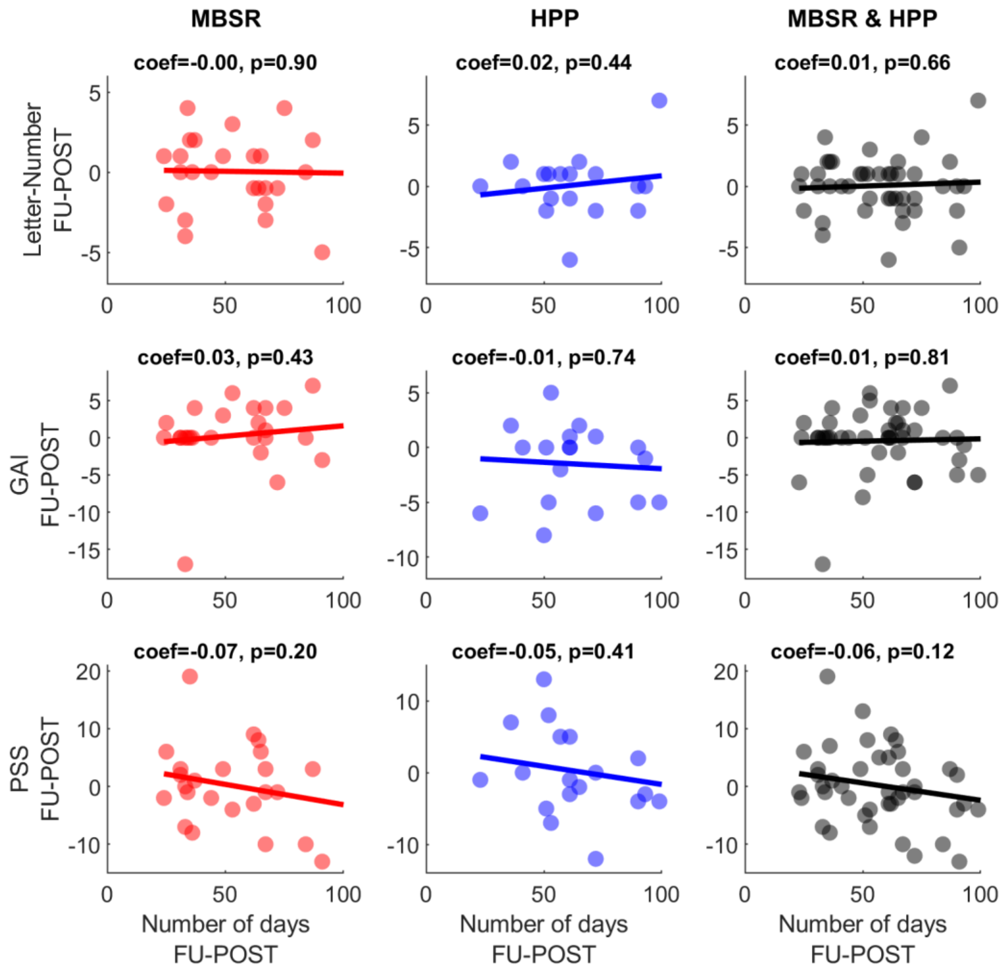

Figure S5.

Linear regression between the number of days between the follow-up (FU) and post-intervention (POST) sessions and the difference in Letter-number Sequencing, Geriatric Anxiety Inventory (GAI), and Perceived Stress Score (PSS) between FU and POST sessions in the Health Promotion Program (HPP, shown in blue), the Mindfulness-Based Stress Reduction (MBSR, shown in red) and collapsed across all subjects from HPP and MBSR (shown in black). Each dot represents an individual with more transparent dots representing fewer overlapping data points, while more opaque dots indicate a higher number of overlapping data points. On top of each subplot, coef indicates the regression coefficient and *p* denotes the corresponding p-value

ADVERSE EFFECTS

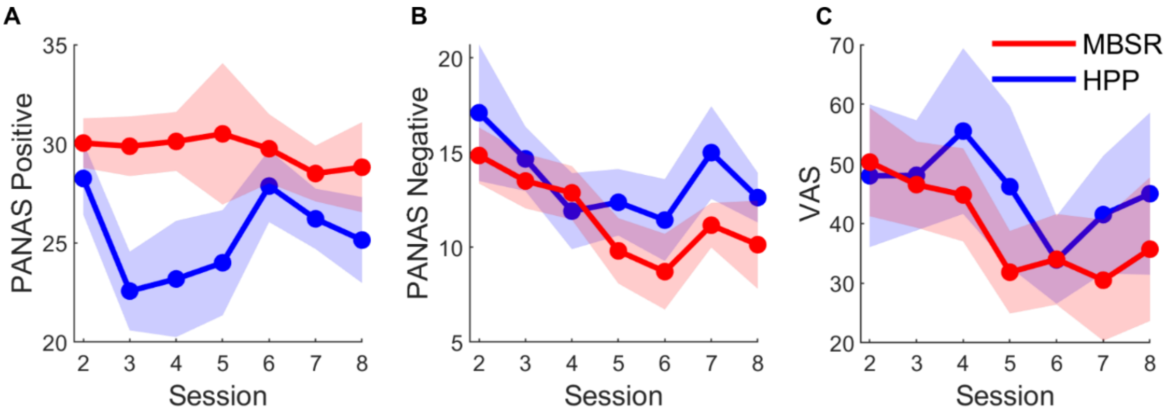

Figure S6.

PANAS and VAS across groups sessions.

The graphs illustrate the change in mean scores across intervention sessions. The questionnaires started to be administered only after the second session. The Health Promotion Program (HPP) is shown in blue, while the Mindfulness-Based Stress Reduction (MBSR) is depicted in red. Shading represents the standard error of the mean.

Table S5

Visual Analog Scale (VAS) and Positive and Negative Affect Schedule (PANAS) Scores Across Sessions

	Session							***Model estimate and p-value***		
	2	3	4	5	6	7	8	Group comparison	Time comparison	Group x Time
**VAS**										
HPP	48.0 (37.8)	48.1 (33.3)	55.5 (43.9)	46.2 (44.8)	33.9 (24.4)	41.5 36.8)	45.0 (30.4)	*3.50 (11.74)*	-1.39(1.16)	-1.76 (1.56)
MBSR	50.3 (28.7)	46.5 (33.0)	44.8 (30.2)	31.8 (24.0)	34.0 (26.3)	30.5 (38.0)	35.7 (38.1)	*p* = 0.77	*p* = 0.23	*p* = 0.26
**PANAS +**									0.08747 0.39537	
HPP	28.2 (5.74)	22.6 (7.42)	23.2 (9.71)	24 (9.92)	27.9 (7.02)	26.2 (5.69)	25.1 (5.73)	4.52 (3.04)	155.28225	-0.21 (0.53)
MBSR	30.0 (4.49)	29.9 (6.00)	30.1 (6.11)	30.5 (14.7)	29.8 (6.03)	28.5 (5.95)	28.8 (7.52)	*p* = 0.14	0.221 0.825	*p* = 0.69
**PANAS -**										
HPP	17.1 (11.4)	14.7 (6.55)	11.9 (6.66)	12.4 (7.01)	11.4 (8.63)	15.0 (9.14)	12.6 (3.70)	2.47 (2.47)	-0.04 (0.28)	**-0.78 (0.38)**
MBSR	14.8 (5.32)	13.5 (6.12)	12.9 (5.64)	9.8 (6.90)	8.71 (7.47)	11.2 (4.99)	10.1 (7.71)	*p* = 0.32	*p* = 0.88	***p* = 0.04***

Abbreviations: PANAS +, Positive and Negative Affect Schedule (positive); PANAS -, Positive and Negative Affect Schedule (negative).

SENSITIVITY ANALYSIS OF PARTICIPANTS WITH HIGH SESSION COMPLETION

In this section, we present the data from the sensitivity analysis, which includes only participants who completed at least 60% of the intervention sessions.

Table S6

Means and Standard Deviations for Primary and Secondary Outcome Measures of Participants Completing at Least 60% of Intervention Sessions

				***Model estimate and p*-value**		
	**BSL**	**POST**	**FU**	Group comparison	Time comparison	Group x Time
**Sample size**						
HPP	n = 16	n = 16	n = 16	-	-	-
MBSR	n = 21	n = 21	n = 21			
**Letter-number**						
HPP	6.06 (2.32)	6.60 (2.90)	6.07 (2.23)	*1.02 (0.85)*	0.03 (0.28)	*0.*16 (0.37)
MBSR	7.10 (2.59)	7.76 (2.95)	7.50 (2.76)	*p* = .24	*p* = .81	*p* = .67
**STROOP**						
HPP	1.23 (7.55)	0.90 (7.02)	2.96 (11.45)	-1.37 (2.93)	0.91 (1.38)	0.09 (1.82)
MBSR	-1.23 (8.05)	2.16 (10.68)	0.70 (9.85)	*p* = .64	*p* = .51	*p* = .96
**TMT**						
HPP	196.33 (146.60)	128.93 (101.61)	149.08 (122.97)	-43.05 (36.92)	-25.70 (15.19)	13.18 (19.86)
MBSR	130.61 (129.17)	136.16 (91.95)	111.55 (72.81)	*p* = .25	*p* = .10	*p* = 0.51
**MMSE**						
HPP	27.81 (1.47)	27.33 (1.23)	27.93 (1.54)	0.03 (0.56)	-0.04 (0.21)	0.19 (0.28)
MBSR	27.67 (2.08)	28.00 (1.87)	28.00 (1.81)	*p* = .96	*p* = .87	*p* = .50
**PSQI**						
HPP	8.19 (3.83)	7.27 (3.88)	7.79 (3.70)	-1.79 (1.22)	-0.37 (0.48)	0.11 (0.63)
MBSR	6.05 (3.44)	6.33 (3.94)	5.35 (4.06)	*p* = .15	*p* = .45	*p* = .86
**GDS**						
HPP	0.75 (1.24)	1.07 (1.22)	0.86 (1.10)	0.13 (0.37)	0.05 (0.18)	-0.15 (0.23)
MBSR	1.05 (1.28)	0.67 (1.02)	0.85 (1.27)	*p* = .73	*p* = .76	*p* = .50
**GAI**						
HPP	9.94 (6.43)	8.60 (6.01)	8.00 (6.29)	-1.36 (2.01)	**-1.25* (0.62)**	-0.52 (0.82)
MBSR	9.00 (6.21)	5.76 (6.40)	5.55 (6.02)	*p* = .50	***p* < .05**	*p* = .53
**PSS**						
HPP	16.20 (9.17)	11.87 (5.94)	12.93 (7.04)	-1.88 (2.33)	-1.94 (1.02)	0.25 (1.33)
MBSR	13.95 (6.61)	11.43 (6.20)	10.60 (8.30)	*p* = .42	*p* < .06	*p* = .85
**MAAS**						
HPP	63.53 (13.75)	68.93 (14.31)	67.21 (10.82)	2.47 (4.07)	2.06 (1.71)	0.22 (2.24)
MBSR	68.65 (12.04)	67.76 (14.09)	73.05 (10.10)	*p* = .55	*p* = .23	*p* = .92

**Cortisol**						
HPP	-6.84 (2849.45)	-673.00 (3029.20)	-	-16.19 (10.67)	-2.52 (4.22)	2.49 (5.54)
MBSR	-332.21 (1809.37)	-424.20 (2297.54)		*p* = .14	*p* = .55	*p* = .65
**HRV**						
HPP	43.24 (59.27)	53.48 (48.24)	37.32 (22.19)	-325.37 (820.80)	-666.16 (888.96)	574.17 (1177.92)
MBSR	28.95 (21.27)	35.35 (19.04)	29.17 (14.94)	*p* = .69	*p* = .46	*p* = .63

Abbreviations: Continuous variables are presented as means (SD), categorical variables as numbers. Abbreviations: HPP, Health Promotion Program; MBSR, Mindfulness-Based Stress Reduction; MMSE, Mini-Mental State Examination; PSQI, Pittsburgh Sleep Quality Index; GDS, Geriatric Depression 5 Item Scale; GAI, Geriatric Anxiety Inventory; PSS, Perceived Stress Score, MAAS; Mindfulness Attention and Awareness Scale; BSL, Baseline; POST, Post-Training; FU, Follow-Up. *$p < .05$.

Reporting checklist for randomised trial.

Based on the CONSORT guidelines.

Instructions to authors

Complete this checklist by entering the page numbers from your manuscript where readers will find each of the items listed below.

Your article may not currently address all the items on the checklist. Please modify your text to include the missing information. If you are certain that an item does not apply, please write "n/a" and provide a short explanation.

Upload your completed checklist as an extra file when you submit to a journal.

In your methods section, say that you used the CONSORTreporting guidelines, and cite them as:

Schulz KF, Altman DG, Moher D, for the CONSORT Group. CONSORT 2010 Statement: updated guidelines for reporting parallel group randomised trials

		Reporting Item	Page Number
**Title and Abstract**			
Title	#1a	Identification as a randomized trial in the title.	1
Abstract	#1b	Structured summary of trial design, methods, results, and conclusions	1-2
**Introduction**			
Background and objectives	#2a	Scientific background and explanation of rationale	3-4
Background and objectives	#2b	Specific objectives or hypothesis	4-5
**Methods**			
Trial design	#3a	Description of trial design (such as parallel, factorial) including allocation ratio.	5-6
Trial design	#3b	Important changes to methods after trial commencement (such as eligibility criteria), with	n/a

		reasons	
Participants	#4a	Eligibility criteria for participants	6-7
Participants	#4b	Settings and locations where the data were collected	5
Interventions	#5	The experimental and control interventions for each group with sufficient details to allow replication, including how and when they were actually administered	8-9
Outcomes	#6a	Completely defined prespecified primary and secondary outcome measures, including how and when they were assessed	9-13
Sample size	#7a	How sample size was determined.	7
Sample size	#7b	When applicable, explanation of any interim analyses and stopping guidelines	
Randomization - Sequence generation	#8a	Method used to generate the random allocation sequence.	7-8
Randomization - Sequence generation	#8b	Type of randomization; details of any restriction (such as blocking and block size)	7
Randomization - Allocation concealment mechanism	#9	Mechanism used to implement the random allocation sequence (such as sequentially numbered containers), describing any steps taken to conceal the sequence until interventions were assigned	7
Randomization - Implementation	#10	Who generated the allocation sequence, who enrolled participants, and who assigned participants to interventions	7
Blinding	#11a	If done, who was blinded after assignment to interventions (for example, participants, care providers, those assessing outcomes) and how.	7-8
Blinding	#11b	If relevant, description of the similarity of interventions	8-9
Statistical methods	#12a	Statistical methods used to compare groups for primary and secondary outcomes	14-15
Statistical methods	#12b	Methods for additional analyses, such as subgroup analyses and adjusted analyses	15

Outcomes	#6b	Any changes to trial outcomes after the trial commenced, with reasons	n/a
**Results**			
Participant flow diagram (strongly recommended)	#13a	For each group, the numbers of participants who were randomly assigned, received intended treatment, and were analysed for the primary outcome	16, Figure 2
Participant flow	#13b	For each group, losses and exclusions after randomization, together with reason	18-19
Recruitment	#14a	Dates defining the periods of recruitment and follow-up	5, Figure 1
Recruitment	#14b	Why the trial ended or was stopped	6
Baseline data	#15	A table showing baseline demographic and clinical characteristics for each group	17, Table 1
Numbers analysed	#16	For each group, number of participants (denominator) included in each analysis and whether the analysis was by original assigned groups	20-21
Outcomes and estimation	#17a	For each primary and secondary outcome, results for each group, and the estimated effect size and its precision (such as 95% confidence interval)	19-22; Table S3 and S4
Outcomes and estimation	#17b	For binary outcomes, presentation of both absolute and relative effect sizes is recommended	n/a
Ancillary analyses	#18	Results of any other analyses performed, including subgroup analyses and adjusted analyses, distinguishing pre-specified from exploratory	23
Harms	#19	All important harms or unintended effects in each group (For specific guidance see CONSORT for harms)	24
**Discussion**			
Limitations	#20	Trial limitations, addressing sources of potential bias, imprecision, and, if relevant, multiplicity of analyses	844-890; 963-974
Generalisability	#21	Generalisability (external validity, applicability) of the trial findings	33

Interpretation	#22	Interpretation consistent with results, balancing benefits and harms, and considering other relevant evidence	24-28
Registration	#23	Registration number and name of trial registry	Title Page
**Other information**			
Protocol	#24	Where the full trial protocol can be accessed, if available	5
Funding	#25	Sources of funding and other support (such as supply of drugs), role of funders	Title page

The CONSORT checklist is distributed under the terms of the Creative Commons Attribution License CC-BY. This checklist was completed on 24. June 2024 using https://www.goodreports.org/, a tool made by the EQUATOR Network in collaboration with Penelope.ai
